# Elevated precipitation alters the community structure of spring ephemerals by changing dominant species density in Central Asia

**DOI:** 10.1002/ece3.6057

**Published:** 2020-02-12

**Authors:** Yangyang Jia, Yu Sun, Tao Zhang, Zhaoyong Shi, Baidengsha Maimaitiaili, Changyan Tian, Gu Feng

**Affiliations:** ^1^ College of Resources and Environmental Sciences China Agricultural University Beijing China; ^2^ Cultivation and Farming Research Institute Heilongjiang Academy of Agriculture Science Harbin China; ^3^ Key Laboratory of Vegetation Ecology Ministry of Education Institute of Grassland Sciences Northeast Normal University Changchun China; ^4^ College of Agriculture Henan University of Science and Technology Luoyang China; ^5^ Institute of Nuclear Technology and Biotechnology Xinjiang Academy of Agricultural Science Urumqi China; ^6^ Xinjiang Institute Ecology and Geography Chinese Academy of Sciences Urumqi China

**Keywords:** Central Asia, dominant species, elevated precipitation, ephemeral community, plant density, rare species

## Abstract

Global climate change is one of the most pressing conservation challenges; in particular, changes in precipitation regimes have already substantially influenced terrestrial ecosystems. However, the mechanisms influencing precipitation changes on individual plants and the plant communities in desert grasslands have yet to be fully elucidated. We therefore examine the influence of increased precipitation on plant community compositions in the Gurbantunggut Desert, Xinjiang, northwestern China, from 2005 to 2009. We found that growth of all plant species and the community productivities increased markedly with enhanced water input. Cover of ephemeral synusia also significantly increased due to increased precipitation, implying that the role of the ephemeral community for stabilization of sand dunes was strengthened by increased precipitation. The response of plant community compositions to increased precipitation was primarily reflected as changes in plant density, while increased precipitation did not affect plant species richness and the diversity index. Dominant species drove the response of plant density to increasing precipitation during the five‐year study period. However, the relative responses of rare species were stronger than those of the dominant species, thereby potentially driving species turnover with long‐term increased precipitation. This finding improved our understanding of how increased precipitation drives the changes in plant community composition in desert grasslands and will help to better predict changes in the community composition of ephemerals under future global climate change scenarios.

## Graphical abstract

Elevated precipitation markedly increased the growth of all plant species. The response of plant community composition to the increasing precipitation was primarily reflected in the changes of plant density. Dominant species drove the response of plant density to the increasing precipitation.

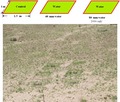

## 1 INTRODUCTION

The earth has experienced substantial precipitation changes in the past five decades, and climate change models predict further changes in precipitation regimes, with increased annual precipitation in many regions (IPCC, 2007). Many studies have demonstrated that global climate change has caused a series of ecological consequences, such as those for plant production (De Boeck, Hiltbrunner, Verlinden, Bassin, & Zeiter, 2018; Zhao et al., 2019) or vegetation changes (Jiang, Bao, Guo, & Ndayisaba, 2017; Li, Chen, Li, Deng, & Fang, 2015). However, changes in the precipitation regime are not uniform and are complicated, revealing spatial and temporal variations that are difficult to study (Li, Zhang, et al., 2015; Walther et al., 2002). Precipitation in the mid and high latitudes of the Northern Hemisphere has generally increased by 0.5 ± 1% per decade during the last 1,000 years in some places (IPCC, 2007). Zhang et al. (2007) showing that anthropogenic forcing has had a detectable influence on observed changes in average precipitation during the twentieth century, including increases in precipitation in the mid‐latitudes of the Northern Hemisphere. In the past century, Central Asia has experienced a complex pattern of precipitation changes (Lioubimtseva & Henebry, 2009). Overall, the Central Asia dry lands may become moister, and extreme precipitation events may increase as a result of global warming due to a probable intensification of westerly cyclones (Deng & Chen, 2017; Lioubimtseva, Cole, Adams, & Kapustin, 2005; Luo et al., 2018). It is therefore important and necessary to better understand how these precipitation changes affect plant community compositions and ecosystem functions in this changing environment.

If it is possible to identify functional characteristics at an individual level that determines the responses of ephemeral plant communities to precipitation change, the potential for predicting outcomes for a range of different situations will be improved (Morecroft et al., 2004). Observational studies have suggested some likely patterns of response. When drought occurs in perennial grasslands, the dominant grass species tend to die first, reducing community productivity and leaving gaps in the sward for other short‐lived plants, followed by increased frequency and abundance of short‐lived plants in the following years (Grime, Willis, Hunt, & Dunnett, 1994). It has been proven that deep‐rooted species tend to increase after drought events (Dunnett, Willis, Hunt, & Grime, 1998). With an increased frequency of drought events, we may see a shift in grassland composition toward more deep‐rooted and short‐lived plants. Seed productivity is also an important factor for determining plant community structure through seed sizes and numbers (Leishman, 2001). Changes in precipitation can significantly affect seed productivity and phenology, especially in harsh environments (Chen et al., 2019). Researchers have found that although decreases in precipitation suppress the aboveground biomass of plant communities, net primary production (NPP) is significantly more sensitive to increased precipitation than to decreased precipitation, especially in arid and semiarid environments (Knapp & Smith, 2001; Zhao et al., 2019). Most experimental manipulations have been performed in perennial grasslands (Byrne, Adler, & Lauenroth, 2017; Dunnett et al., 1998), and the Central Asia arid area is one of the largest arid regions at middle latitudes, and includes approximately one‐third of the arid lands and almost 90% of the temperate deserts worldwide (Lioubimtseva & Henebry, 2009), but research on the effects of precipitation changes on plant communities in this area are rare (Zhang, Li, Zhang, Zhang, & Chen, 2016).

Water availability is the most important factor determining plant growth and primary productivity in all ecosystems, especially in arid and semiarid grasslands (Dube & Pickup, 2001; Nielsen, Osler, Campbell, Burslem, & Van Der Wal, 2010). Recent studies in Central Asia have shown that increased precipitation is beneficial for the growth of *Haloxylon ammodendron* (Zhao et al., 2019); significantly improves the growth and seed production of annual desert plants (Chen et al., 2019); and the dynamics of vegetation cover are closely related to precipitation changes (Zhang, Lu, et al., 2016). However, plant community structures may respond more strongly to increased precipitation than to drought, because plants are more drought‐tolerant in arid ecosystems (Knapp & Smith, 2001). In addition, the responses of plant communities to changes in precipitation regimes rely greatly on community structures and plant species compositions (Gerten et al., 2008; Suding et al., 2008; Torode et al., 2016). On one hand, precipitation changes, for example, a long‐term precipitation increase in grasslands, cause difficulty in obtaining consistent effects for community diversity, because of the high variations across survey years (Collins et al., 2012; Yang et al., 2011). On the other hand, the dominant species in the plant community determine the responses of the plant community to changes in precipitation (Smith, Knapp, & Collins, 2009). If the dominant plant species respond strongly to climate change, then significant changes in the plant community can be expected (Byrne et al., 2017). However, the dominant species in arid lands may be less responsive to increased precipitation because they may already have adapted to the arid environment (Harpole & Tilman, 2007). Therefore, although it is clear that precipitation changes have significant effects on plant communities (NPP), we still have an incomplete understanding how plant communities respond to increased precipitation in arid and semiarid grasslands.

Spring ephemerals are widely distributed in the arid lands of Central Asia, and precipitation is more important than soil chemistry in determining the distribution of ephemerals (Zhang, Liu, Zhang, & Sun, 2016). These spring ephemerals offer an intriguing survey opportunity to observe their responses to increased precipitation. In this area, each year supports a new generation of ephemerals experiencing climates that differ from year to year because ephemerals are very sensitive to precipitation changes (Zhang & Chen, 2002). Thus, only by multiyear sampling can we capture several unique combinations of plant community properties and environmental conditions. Although the lifecycles of spring ephemerals are only 60–70 days and finish before the summer drought arrives (Zhang, 2002), they play important ecological roles in Central Asia. For instance, in early spring, although their biomass may be less than that of shrubs in most arid ecosystems, ephemerals cover the spaces between shrubs and can greatly decrease the frequency and intensity of sandstorms. Moreover, ephemerals are the major components of transitory desert grasslands, and provide forage for livestock in early spring when there are few other resources available (Zhang, 2002). A change in any of these functions is significant for ecosystem stability in Central Asia, but predicting the response of this highly functional vegetation component to increased precipitation first requires a multiyear survey. Compared to perennial grassland ecosystems, the potential influences of climate change on the compositions of ephemeral plant communities are poorly understood. The aim of this study is to examine how and to what extent increased precipitation changes ephemeral plant communities and further alters ecosystem functions. We hypothesized that (a) increased precipitation was positive for changes in community structure, increasing NPP and plant cover, and for enhancing ecosystem functions; (b) the responses of the dominant species decided the degree of response of the plant community, and rare species had little effect. To test this hypothesis, a five‐year field trial was conducted from 2005 to 2009 in a desert rangeland in the Gurbantunggut Desert, northwestern China.

## 2 MATERIALS AND METHODS

### 2.1 Study site

The Gurbantunggut Desert, a fixed and semifixed desert, is located in the hinterlands of the Dzungaria Basin in Xinjiang, northwestern China. The mean annual temperature, mean growth season precipitation, and mean annual precipitation are 7.1°C, 67.9 mm, and 215.6 mm, respectively (meteorological datasets are from the Fukang station of the Desert Ecology Chinese Academy of Sciences). During winter, the desert is usually covered by more than 20 cm of snow. Annual evaporation in this region is more than 2,000 mm (Sun & Yang, 2010). Soils are gray desert soils (Chinese classification) with aeolian sands on the surface (0–100 cm; Chen, Wang, Li, & Ruan, 2007). The vegetation consists of a mixture of shrubs and grasses. *Haloxoylon persicum*, which grows on the tops of dunes, is the constitutive species in the desert community. *Ephedra distachya*, a small shrub, is distributed in interdunal valleys. Annual herbs are distributed in the understory of the shrubs (Zhang, 2002).

In spring, ephemeral plants are dominant in the community (Zhang & Chen, 2002). These plants are able to take advantage of the favorable soil moisture and thermal conditions in the spring (around March) to grow and finish their lifecycles within approximately 60–70 days before the summer drought arrives (Zhang, 2002). The ephemerals play a very important role in the stabilization of sand dunes and in reducing the frequency and intensity of sandstorms (Wang, Wang, Jiang, & Zhao, 2005).

### 2.2 Experimental design

Our experimental site is located at N44°32.407′, E88°16.779′ and is in a valley between dunes with an elevation of 510 m. The site is isolated by a steel fence to prevent grazing by animals. A randomized block field experiment was performed continuously from 2005 to 2009. The properties of the sandy soil in the experimental site are shown in Appendix 1.

The experiments involved several irrigation levels. According to the results of recent publications, precipitation will continuously increase by approximately 50 mm in the next 50–100 years (Takayabu et al., 2007; Zhao, Ding, Xu, & Zhang, 2003), and precipitation fluctuations may become as great as +40% in extreme precipitation years (Liu et al., 2010); therefore, we set 40 mm of water as the minimum water input. In 2005, 2007, 2008, and 2009, the treatments consisted of natural rainfall (as control) and 40 mm of water addition based on natural rainfall. In 2006, an additional treatment of 80 mm water was added to simulate future extreme wet years. Usually, water additions were accomplished with a sprinkler kettle, and irrigation was over the entire plot area. The research area was divided into five zones, and, in each year, one zone was harvested to avoid the effects of destructive harvesting on the results. Each zone consisted of sixteen 1 × 1.5 m^2^ plots (in 2006, there were twenty‐four plots), and each treatment had eight replicates. The plots were arranged in a randomized block, each plot was separated from its adjacent plots by a 1 m buffer strip. All plots were divided into a central zone of 1 × 1 m^2^ to investigate plant community changes and a surrounding buffer zone to reduce edge effects from the surrounding untreated vegetation (Appendix 3).

Simulations of increased precipitation started in late March (approximately the 25th of March) of each year before plant emergence and harvesting took place in late May (approximately the 25th of May). Water was input every two weeks after the start of the experiment, for a total of four times during the plant growth season for each of 5 years. The volume of water input each time in each plot was 15 L for the 40 mm water treatment; this is equivalent to a one‐time increase in precipitation of 10 mm, and 30 L was used for the 80 mm treatment; this is equivalent to a one‐time increase in precipitation of 20 mm. Each plot was divided into a central zone of 1 m^2^ and a surrounding buffer zone to reduce marginal utility.

### 2.3 Sampling and analysis

The cover for each plant species and for the entire community, and the plant densities of every species and for entire plots were measured before harvest. Diversity indices, for example, species richness, the Shannon–Weiner index, and the relative abundance of each plant species, were calculated. When most plants were senescing at the end of May (approximately two months after starting), all plants in the plots were dug up. Each plant was taxonomically identified, seed numbers were recorded (this was only done in 2006 and 2007), and each plant was divided into shoots and roots and later dried at 80°C in an oven. The dry weights of each species were recorded.

Species richness was represented by the total number of species per plot; the relative abundance of each species was calculated as the percentage of plots containing that species. The biodiversity index and frequency of appearance were calculated by the Shannon–Weiner index as follows:

$$\text{Shannon}\;-\;\text{Weiner index}=-\text{sum}\;(P_{i}\ln\left[P_{i}\right]),$$

where *P_i_* = *n_i_*/*N* and *n_i_* = number of individuals for species *i*, and *N* = total number of individuals in all species.

Frequency of appearance (%) = Number of plots in which a species emerged during three experimental years × 100/total number of plots in 3 years.

To determine the responses of different plants to experimental water additions, we calculated the differences and response ratios of the plant density responses to water addition for each block as follows:

$$\text{The difference}=T_{m}-C_{m},$$

$$\text{The response ratio}=\left(T_{m}-C_{m}\right)/C_{m},$$

where *T*_m_ is the plant density for the 40 mm or 80 mm water addition treatment plots in block m, and *C*_m_ is the plant density for the control plot in block m.

A total of 37 plant species were observed across all plots during the 5 years of the experiment (Appendix 2). Among these plant species, one species, for example, *Ephedra distachya*, is a small shrub, six are perennials, and six are unidentified, while 24 are ephemeral plants. A species was classified as “dominant” if its frequency was >70% and its relative abundance was >5%. A species was classified as “rare” if its frequency was <50% and its relative abundance was <3% (Ma et al., 2017; Mariotte, Vandenberghe, Kardol, Hagedorn, & Buttler, 2013). This method yielded six ephemeral dominant species, namely *Erodium oxyrrhynchum*, *Ceratocarpus arenarius*, *Carex physodes*, *Alyssum linifolium*, *Hyalea pulchella*, and *Trigonella arcuata*, and 26 rare species (Appendix 2).

### 2.4 Statistical analysis

An a priori structural equation model (SEM) was applied to test the effects of increased precipitation on alterations of the community structures of spring ephemerals, according to hypothesized causal relationships, using R version 3.2.2 (R Foundation for Statistical Computing, Vienna, Austria, 2013), with the “lavaan” package. We selected unique attributes of the plant community: aboveground biomass (ANPP), species richness, plant density, and community structure in our model. To use community structure as a variable in the model, we used a nonmetric multidimensional scaling (NMDS) analysis of plant density, based on the Bray–Curtis distance measure (McCune, Grace, & Urban, 2002), and selected Axis 1 as the community structure in the model. In our NMDS analysis, Axis 1 and Axis 2 explained 41% and 28% of the total variance in the plant community, respectively (stress = 0.2218). We used the *χ* ^2^ test, root mean square error of approximation (RMSEA), and Akaike information criteria (AIC) to evaluate the model fits. Nonmetric multidimensional scaling (NMDS) analyses of plant densities were also performed to examine the effects of increased precipitation on the plant community structures over 5 years.

All data were subjected to a one‐way or two‐way ANOVA using the SPSS17.0 software package for Windows (version 17.0; SPSS Inc.). Treatments were compared using the least‐significant different (LSD) test at *p* < .05. All figures were created with SigmaPlot 10.0.

## 3 RESULTS

### 3.1 Effects of elevated precipitation on ANPP, cover, and seed production

From 2005 to 2009, the climatic regimes of the study site showed typical bell‐shaped curves of precipitation and air temperature, and the precipitation regimes were markedly different (Appendix 4). During the growth season of the ephemeral plants (March to May), precipitation was 29.9, 39.0, 73.2, 48.0, and 88.2 mm from 2005 to 2009, respectively, and the growth season precipitation for 2007 and 2009 was more than double that for 2005, 2006, and 2008. In general, 2007 was wetter than other 4 years (Appendix 1).

Water additions promoted the growth and significantly increased the ANPP of the plant community, but with strong interannual variability across the five study years (*F*_4, 40_ = 20.048, *p* < .0001, Table 1). The greatest increase was in 2006, and the 80mm water treatment significantly increased the biomass of ephemerals by 326%; the smallest increase was in 2008, and the biomass associated with the 40mm water treatment was only significantly higher (22%) than that of the control (Table 2).

**Table 1:** Results of two‐way ANOVA on the effects of year (Y), elevated precipitation (W), and their interactions on the ANPP, coverage, species richness, Shannon–Wiener index, plant density, N uptake, P uptake, K uptake, and seed number

Variable	Source of variation					
	Year (Y, *df* = 4)		Elevated precipitation (W, *df* = 1)		Y × W (*df* = 4)	
	*F*	*p*	*F*	*p*	*F*	*p*
ANPP	20.048	**<.0001**	35.088	**<.0001**	6.892	**<.0001**
Coverage	53.795	**<.0001**	42.449	**<.0001**	10.106	**<.0001**
Species richness	26.358	**<.0001**	0.167	.684	1.414	.238
Shannon–Wiener index	9.903	**<.0001**	0.006	.937	1.841	.131
Plant density	41.269	**<.0001**	3.145	**<.001**	0.649	**<.001**
Seed number	21.833 (*df* = 1)	**<.0001**	0.532	**<.001**	0.283 (*df* = 1)	**<.001**

Note

*P*‐values in bold are significantly different (*p* < .05).

Degrees of freedom (*df*), *F*‐test values, and *p*‐values are given. Seed number was only tested in 2006 and 2007, so the *df* of Y and Y × W was 1.

**Table 2:** Response of ANPP, seed production, plant density, species richness, and plant diversity index of the desert ephemeral community to input of water from 2005 to 2009

Treatments (mm)	ANPP (g/m^2^)	Seed number of per plot (No.m^−2^)	Plant density (No.m^−2^)	Species richness (species/m^2^)	Shannon–Wiener index (*H′*)
2005					
0	26.1 ± 4.1b	Not measured	117.0 ± 10.5b	8.4 ± 0.9	1.31 ± 0.2
40	41.0 ± 3.5a	Not measured	195.6 ± 11.7a	9.6 ± 0.8	1.51 ± 0.1
2006					
0	57.8 ± 6.9c	3,050 ± 894b	106.7 ± 13.3b	10.5 ± 0.4	1.29 ± 0.1
40	125.0 ± 13.0b	12,156 ± 2,700a	175.0 ± 21.4a	10.6 ± 0.6	1.37 ± 0.1
80	222.1 ± 39.2a	14,593 ± 4,752a	202.3 ± 28.9a	11.5 ± 0.5	1.41 ± 0.1
2007					
0	85.3 ± 14.3b	2,930 ± 286b	243.5 ± 26.0b	13.3 ± 1.2	1.71 ± 0.1
40	145.7 ± 25.2a	5,772 ± 497a	364.6 ± 31.7a	14.1 ± 1.5	1.80 ± 0.1
2008					
0	83.5 ± 8.9b	Not measured	198.0 ± 28.9b	12.6 ± 0.7	1.33 ± 0.1
40	102.0 ± 8.4a	Not measured	322.0 ± 30.1a	14.1 ± 0.7	1.49 ± 0.1
2009					
0	102.6 ± 10.0b	Not measured	453.5 ± 40.0b	18.0 ± 0.8	1.78 ± 0.1
40	285.5 ± 45.0a	Not measured	626.4 ± 75.3a	17.5 ± 0.7	1.58 ± 0.1

Note

Data were expressed by the mean ± *SE*. The values within a column marked by different letters represented significant difference at *p* < .05.

**Figure 1:**
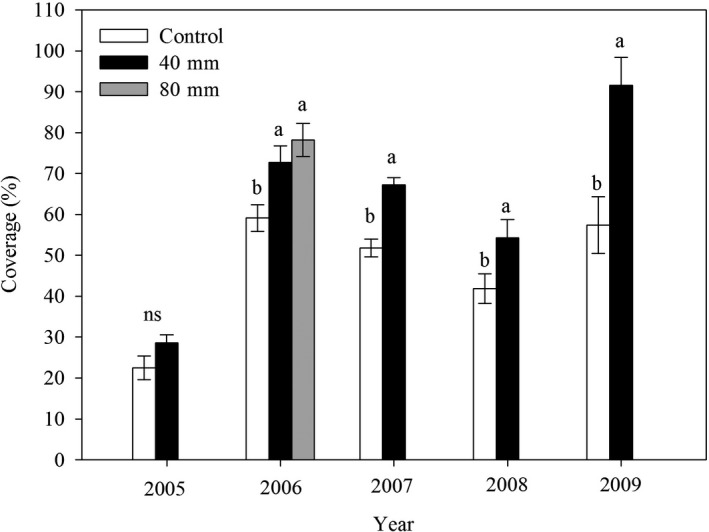
Response of the coverage of plant community to water input. Columns marked with different letters indicated significant differences (*p* < .05) between treatments. Error bars represented ± *SE*

The ephemeral cover varied in different years (*F*_4, 40_ = 53.795, *p* < .0001, Table 1), and significantly increased with increased precipitation (*F*_1, 40_ = 42.449, *p* < .0001, Table 1). There were no significant differences between the two treatments in 2005 (Figure 1). The largest increase was in 2009, and the 40mm water treatment significantly increased the cover by 59% (Figure 1).

Elevated precipitation significantly promoted seed production of the ephemeral plants(*F*_1, 16_ = 0.532, *p* < .001, Table 1). In 2006, the 40 and 80 mm water treatments significantly increased seed production by 298% and 378%, respectively, compared to the control. No differences in seed production were observed between the water addition treatments of 40 and 80 mm. In 2007, seed production associated with the 40mm water treatment was 97% higher than that of the control (Table 2). Elevated precipitation had no significant effects on species richness and the Shannon–Wiener index across the 5 years (Tables 1 and 2).

### 3.2 Effects of elevated precipitation on the proportion of ANPP and seed production of dominant species

**Figure 2:**
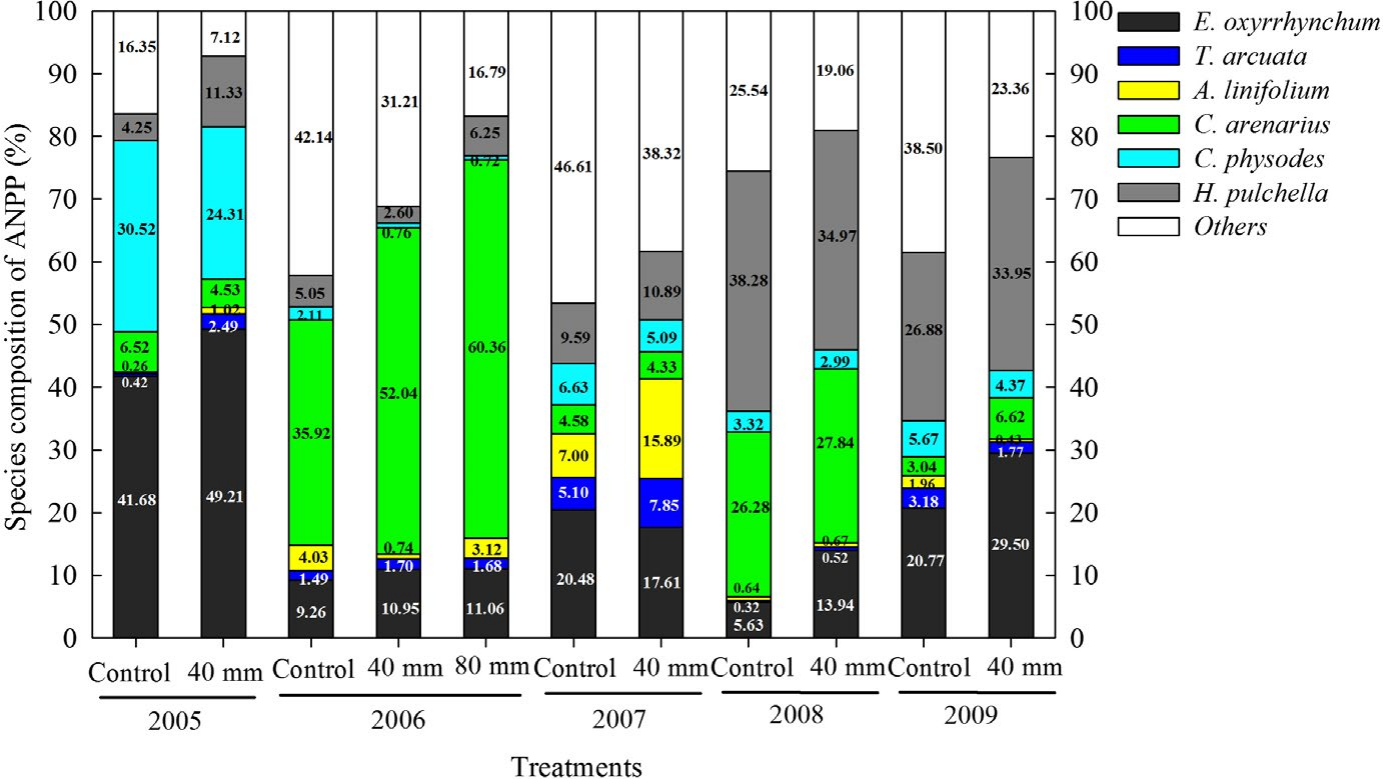
Contribution of dominant plant species to the ANPP between different treatments from 2005 to 2009. Data were the mean of eight replications

Water addition significantly increased the ANPP of the plant community, but the responses of individual species varied considerably between different treatments (Figure 2, Appendix 5). In 2005, the relative abundance of *E. oxyrrhynchum* was greatest, and significantly increased by 18% with the 40mm water addition treatment compared to the control. *H. pulchella* abundance significantly increased by 167%. *C. physode* was the second most abundant species, but it was significantly reduced by 20%. In 2006, *C. arenarius* was the most abundant species, and water additions significantly increased growth, but there was no significant difference between the 40mm and 80mm water treatments. In 2007, water additions had no significant effects on plant growth, except to significantly increase the proportion of *A. linifolium*. *H. pulchella* was the most abundant species, but there were no significant differences between the two treatments both in 2008 and 2009. However, water additions significantly increased the relative abundances of *E. oxyrrhynchum* by 148% and 42% in 2008 and 2009, respectively. Overall, water additions increased the total proportions of the dominant species across 5 years (Figure 2).

**Figure 3:**
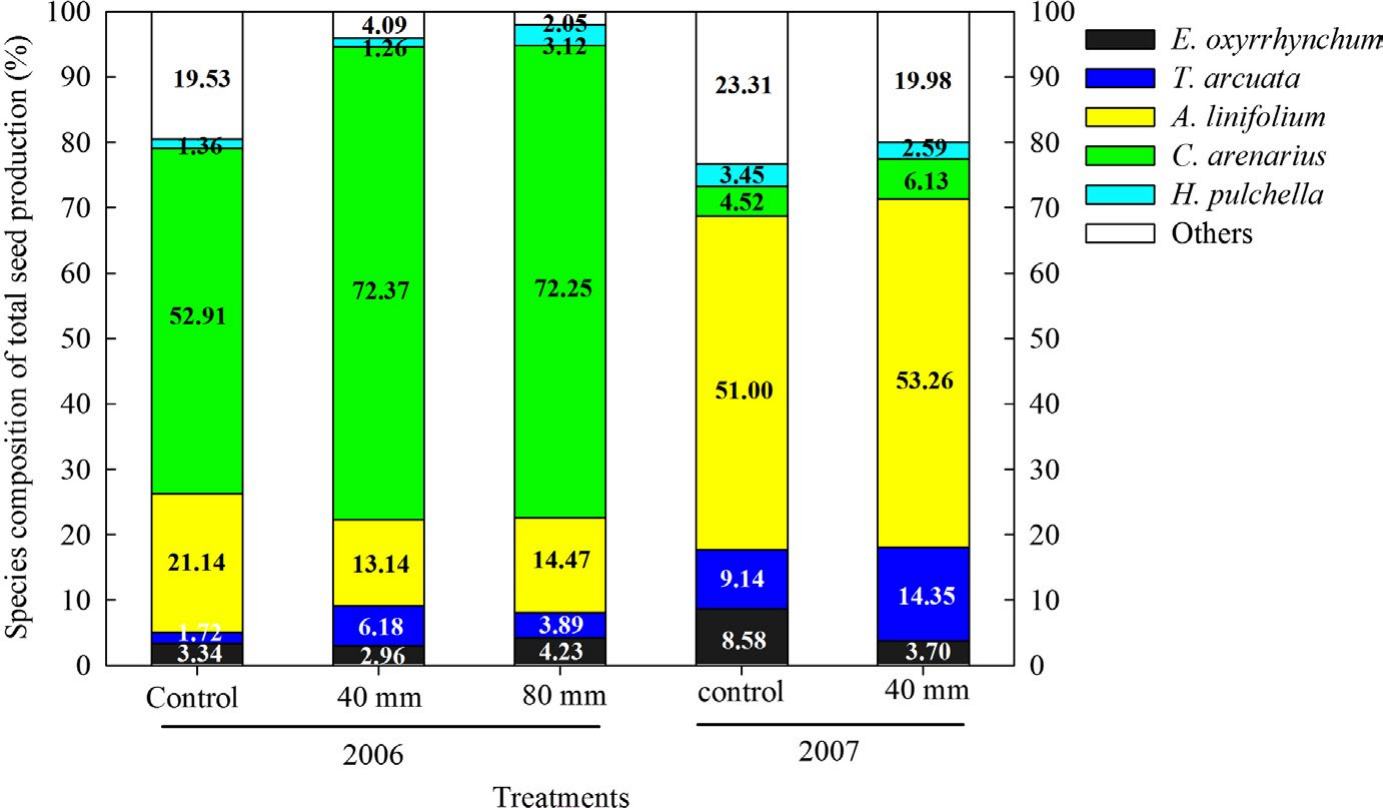
Contribution of dominant plant species to the total seed production between different treatments in 2006 and 2007. Data were mean of eight replications

Water additions altered the relative contributions of each species to seed production both in 2006 and 2007 (Figure 3). Water additions resulted in a significant increase in the proportions of seed production generated by *T. arcuata* and *C. arenarius* in 2006 and 2007. However, water additions resulted in 37% (*p* < .05) and 32% (*p* < .05) reductions in the proportions of total seed production generated by *A. linifolium* for the 40 and 80 mm water additions, respectively, in 2006. In 2007, water additions reduced the seed proportion of *E. oxyrrhynchum* (*p* < .05). Water additions had no effects on the seed productions of *H. pulchella* in 2 years or on the seed production of *E. oxyrrhynchum* in 2006 (*p* > .05). In general, compared to the 40 mm water addition, the 80 mm addition had no effect on seed production for the five dominant species. However, both water additions increased the total proportions of seed production for the five dominant species in the 2 years (Figure 3).

### 3.3 Effects of elevated precipitation on the community composition of ephemerals

**Figure 4:**
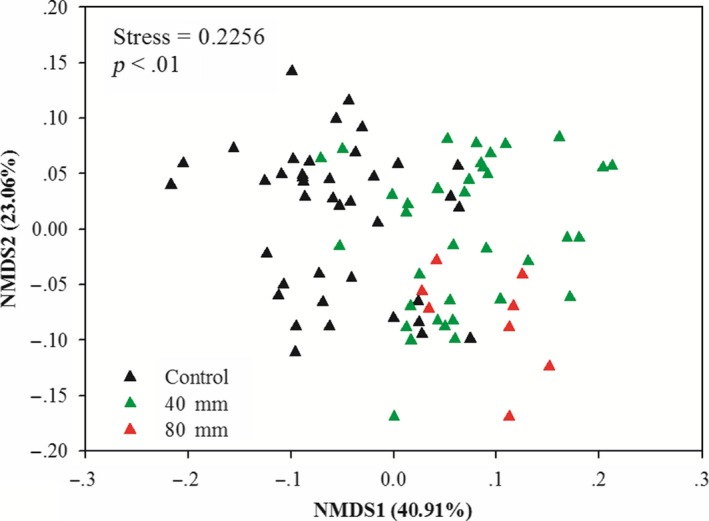
Nonmetric multidimensional scaling (NMDS) ordination of plant community composition in response to increasing precipitation across 5 years In the plots, black triangles, control; green triangles, 40 mm; red triangles, 80 mm (only in 2006)

Plant density was significantly enhanced by water additions compared to the control at harvest time from 2005 to 2009 (*F*_1, 40_ = 3.145, *p* < .001, Table 2). The highest increase was in 2005, and the 40mm water addition significantly increased plant density by 79%; the smallest increase was in 2009, and the 40 mm water addition significantly increased plant density by 38%. In 2006, the plant density for the 80 mm water addition was 190% higher than that of the control, but no difference was observed between the 40 and 80 mm water addition treatments. Plant densities were significantly different across 5 years (*F*_4, 40_ = 41.269, *p* < .0001, Table 1), and there were interactive effects on plant density (*F*_1, 40_ = 0.649, *p* < .001, Table 1). The arrangement of plant species in the ordination diagram (NMDS, Figure 4) clearly reflects the divergence in plant community compositions caused by increased precipitation.

**Figure 5:**
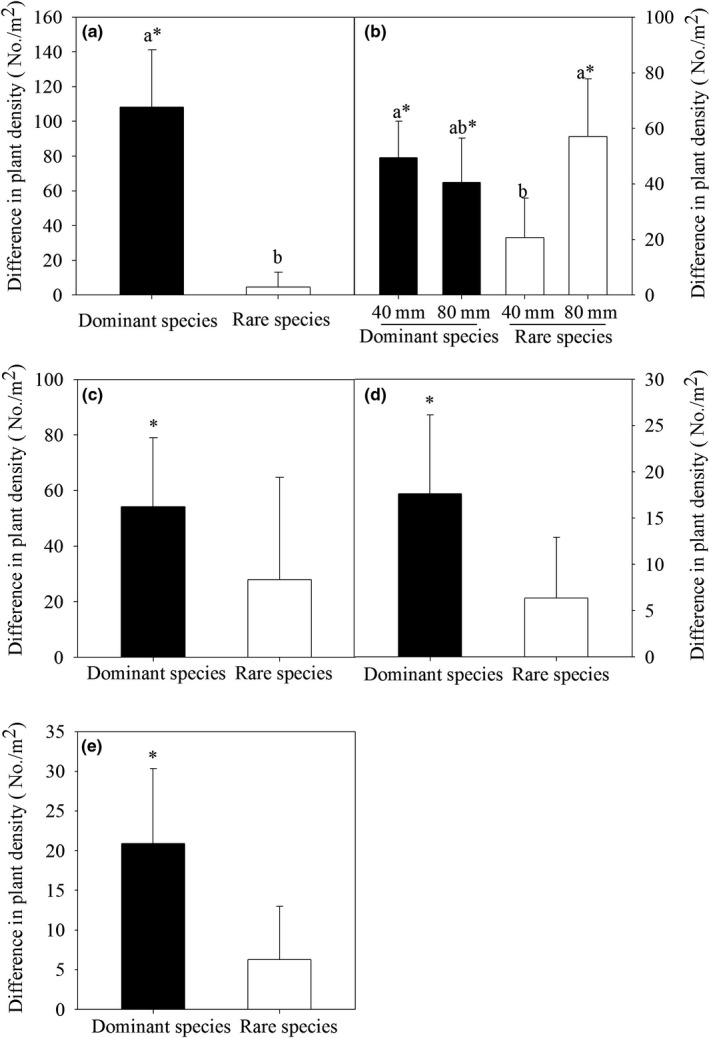
The difference of plant density of two vegetation types (dominant species and rare species) to water addition from 2005 to 2009 (a, 2005; b, 2006; c, 2007; d, 2008; e, 2009). In 2006, we calculated the difference of 40 and 80 mm addition treatments to the control. Significant differences in response to the water addition treatments between the dominant and rare species are indicated by the lowercase letters. Significant differences from zero are indicated with asterisk. Shown are mean ± *SE*

In addition, we combined the plant species to form dominant species group and a rare species group, and these groups responded differently to water additions from 2005 to 2009 (Figure 5). The differences in plant density between the treatment and control plots differed significantly between vegetation types, with the dominant species exhibiting greater responses to water addition than the rare species, and the rare species exhibited no response to water addition, except in 2005 (Figure 5a). In 2006, the difference for the dominant species under the 80 mm treatment responded significantly to water addition, but there was no difference between the 40 mm and 80 mm water addition treatments. The dominant species under the 40 mm treatment exhibited a greater response to water addition than the rare species, but the difference for rare species was even greater than the difference for dominant species under the 80 mm treatment, although this difference was not significant. The difference of the rare species under the 80 mm treatment was higher than for the 40mm water addition treatment (Figure 5b).

**Figure 6:**
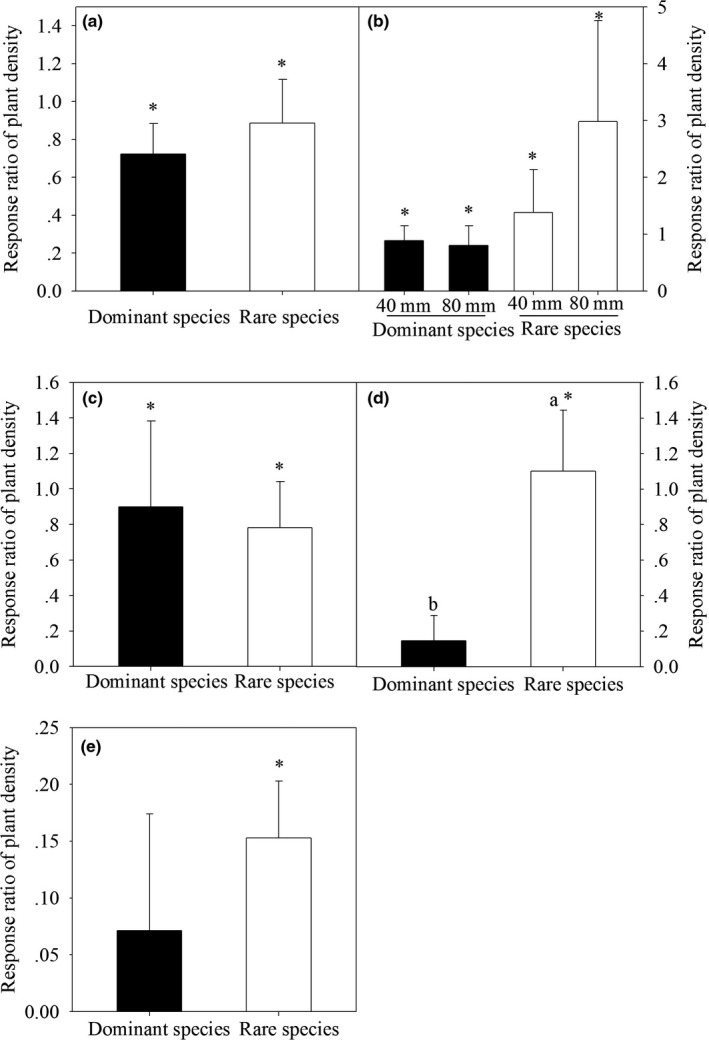
The response ratio of plant density of two vegetation types (dominant species and rare species) to water addition from 2005 to 2009 (a, 2005; b, 2006; c, 2007; d, 2008; e, 2009). In 2006, we calculated the response ratio of 40 and 80 mm addition treatments to the control. Significant differences in response to the water addition treatments between the dominant and rare species are indicated by the lowercase letters. Significant differences from zero are indicated with asterisk. Shown are mean ± *SE*

All response ratios of the two vegetation types responded significantly to water addition, except the dominant species in 2009 (Figure 6). The response ratios of the rare species were greater than those of the dominant species, except in 2007 (Figure 6c), but were only significant in 2008 (Figure 6d).Additionally, the response ratios of the rare species under the 80mm water treatment were greatest in 2006 (Figure 6b).

**Figure 7:**
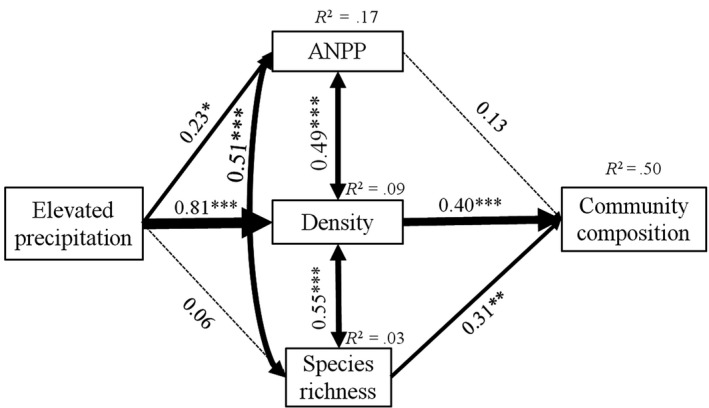
A structural equation model of increasing precipitation, ANPP, density, and species richness as predictors of ephemeral community composition. Solid lines indicate significant path coefficients and dashed lines indicate nonsignificant path coefficients. Bold numbers indicate the standard path coefficients. Arrow width is proportional to the strength of the relationship. *R* ^2^ represent the proportion of variance explained for each dependent variable in the model. ***p, .001; **p, .01; *p, .05; *χ* ^2^ = 0.047, *p* > .05, root mean square error of approximation (RMSEA) = 0.000, Akaike information criteria (AIC) = 904.006

The SEM explained the relationships among ANPP, plant density, species richness, and community composition under elevated precipitation, and fit the data well (Figure 7). ANPP, plant density and species richness were significantly related to each other. Elevated precipitation altered community compositions mainly by affecting plant densities (*λ* = 0.81). Although elevated precipitation significantly affected ANPP (*λ* = 0.23), ANPP had no effects on the community compositions (Figure 7).

## 4 DISCUSSION

Research regarding the change mechanisms of plant community compositions and ecosystem functions in natural ecosystems is always a major research challenge in ecology (Kane et al., 2017; Xu et al., 2018), especially in response to climate changes in recent decades (Eskelinen & Harrison, 2015). Desert ecosystems are more dependent on water availability than on any other factor (e.g., nutrients) in arid and semiarid regions (Weltzin et al., 2003). Precipitation in the arid regions of Central Asia, such as the Dzungaria Basin, has increased by 20% following global climate changes in recent decades (Li, Zhang, et al., 2015) and seems likely to increase by approximately 50 mm in the future (Takayabu et al., 2007; Zhao et al., 2003), and the precipitation regime has changed (Lioubimtseva & Henebry, 2009). The effects of changes in precipitation on ecosystem processes, such as vegetation dynamics (Petrie et al., 2018) and plant productivity (Li, Zhang, et al., 2015) in desert ecosystems have been a concern in recent years (Jiang et al., 2017; Muldavin, Moore, Collins, Wetherill, & Lightfoot, 2008; Thomey et al., 2011). Understanding the ecological responses of plants to changes in precipitation at the population and community levels is essential for predicting how vegetation will function in the future. The present study showed that enhanced water inputs strongly changed some important ecological processes in the Gurbantunggut Desert, for example, community regime and seed productivity.

### 4.1 Effects of elevated precipitation on plant community structures

The stability and resilience of ecosystems are determined by both the richness and diversity of species (Oliver et al., 2015) and by the community structure (Dieleman, Branfireun, McLaughlin, & Lindo, 2015). Our present study showed that the density, cover, and aboveground biomass of some plant species had a strong positive response to enhanced water addition, while some other species exhibited less‐pronounced changes. These impacts caused significant changes in the community structure, although the species richness and biodiversity index were unchanged over the five‐year duration of our study.

In the present study, strong interannual variations in the measured variables were observed across entire experimental years (Table 1; Figure 1). Strong year‐to‐year fluctuations in plant community parameters, including plant cover and ANPP with annual precipitation have been reported for different grassland ecosystems (Heisler‐White, Knapp, & Kelly, 2008; Yang et al., 2011). Water availability limits plant growth and primary productivity in all ecosystems, especially in arid and semiarid grasslands (Nielsen et al., 2010). It has been reported that ephemerals are sensitive to precipitation changes, and March‐to‐May precipitation is mainly responsible for biomass fluctuations in the same region (Zhang & Chen, 2002; Zhang, Liu, et al., 2016). Thus, the strong interannual variability in the measured variables could have been largely attributed to the year‐to‐year fluctuations in precipitation. In addition, we found that the effects of increasing precipitation on ANPP and cover varied with year and were not in agreement with the fluctuations in growing season precipitation for some years (Figure 1 and Table 2). For example, the growing season precipitation in 2005 (29.9 mm) was only 33.9% of that in 2009 (88.2 mm), but ephemeral cover significantly increased by 59% in 2009 under the 40 mm water treatment, while insignificant effects of increased precipitation were detected in 2005 (Figure 1). This is possibly because the magnitudes of the precipitation increases induced variations that could be mainly attributed to the year‐to year‐changes in soil moisture induced by increasing precipitation, as explained by Yang et al. (2011). Our results are in good agreement with Yang et al. (2011) and Liu, Zhang, and Wan (2009).

In our desert plant community, water additions significantly increased plant densities in the community (Table 2), and the dominant species responded most strongly in terms of absolute plant density to water additions over 5 years (Figure 5). This finding is due to the higher initial densities of the dominant species in every plot. Because the dominant species had higher initial densities, they were better able to respond to precipitation changes in an absolute sense. Studies of other low‐productivity grasslands have also demonstrated that the dominant species responded more to fertilization or disturbance than did the subordinate species (Houseman, Mittelbach, Reynolds, & Gross, 2008), and the aboveground biomass of the dominant species in the grasslands of central North America responded strongly and drove the ANPP of the plant community response to increased nutrient availability (La Pierre, Blumenthal, Brown, Klein, & Smith, 2016). The rare species in our plant community were, by definition, less abundant than the dominant species; therefore, the rare species exhibited lower absolute responses to the experimental water additions than did the dominant species.

However, in the present study, the rare species exhibited almost the same response ratios in 2007 and even had higher relative response ratios to the experimental water additions than the dominant species in the other 4 years (Figure 6). This result indicates that rare species may perform better than dominant species relative to their initial densities in the community. These differences between rare and dominant species may be due to individual traits or to the limitation of the dominant species by other resources (e.g., space or nutrient availability) and thus reduced their potential to respond disproportionately to their initial densities (Harpole & Tilman, 2007; La Pierre et al., 2016). Thus, the greater response ratios of the rare species compared to those of the dominant species in our research provided more evidence that a priority effect limits the responses of rare species to resource limitations (La Pierre et al., 2016). The absolute responses of the rare species in the 5 years of study did not drive the plant density of community response to water additions due to of their low densities in the community. However, we should not ignore the important roles of rare species in the plant community. With long‐term water additions, rare species may become the dominant species when they overcome the priority effect (Avolio et al., 2014). For example, in 2006, the absolute responses of the rare species to the 80mm water addition treatments were higher than those of the dominant species in the plant community, although there was no significant difference; there was a trend in which the absolute response of the rare species was higher when there was greater water input. Once this species turnover occurs, the current rare species will be those that drive the community response to water additions (La Pierre & Smith, 2014).

### 4.2 Effects of elevated precipitation on the soil seed bank

The soil seed bank represents important propagule pools for plant population dynamics (Maighal, Salem, Kohler, & Rillig, 2016) and plays a crucial role in the ecology of different plants (Bekker et al., 1998). Therefore, the soil seed bank is an important component for understanding plant community compositions and ecosystem functions. The composition of the seed bank can also be altered by changes in the contributions of seed yield by individual plant species due to the different responses of seed productivity to water additions (Walck, Hidayati, Dixon, Thompson, & Poschlod, 2011). Based on our results, the annual total seed amounts of the desert community can be significantly changed by enhancing the water supply (Table 1). The size of the soil seed bank influences plant population dynamics, with a larger soil seed bank causing a greater probability for a larger plant community and later changes in the species compositions and community diversities (Rosbakh et al., 2017; Walck et al., 2011). Such results suggest that changes in precipitation might potentially alter the stability and productivity of the desert ecosystem and then influence ecosystem functions.

### 4.3 Effects of elevated precipitation on ecosystem functions

Desert ephemerals form a significant community (Zhang, Lu, et al., 2016; Zhang & Chen, 2002) and are widely distributed in both arid woodlands and open desert areas (Robinson, 2004). Ephemerals play a very important role in the stabilization of sand dunes and in reducing the frequency and intensity of sandstorms (Wang et al., 2005). Moreover, ephemerals are major components of desert grasslands, which provide high‐quality forage for livestock in early spring and, during this time, there are few other grass species available (Zhang & Chen, 2002). Zhang (2002) reported that the biomass of ephemerals in the Gurbantonggut Desert accounts for 58 percent of the total biomass in the community of *Haloxoylon persicum* in precipitation‐rich years. From the present study, changes in the community structure of ephemerals induced by increased water may induce changes in several ecosystem functions:

First, an increase in vegetation cover reduces wind erosion and mitigates the desertification processes. Wind erosion is a leading factor for land desertification and sandstorm disasters and is one of the most serious environmental problems in the world (Guo, Huang, Dong, Van Pelt, & Zobeck, 2014). Many studies have indicated that planting vegetation is an effective measure for control of wind erosion and that vegetation cover was the most important factor (Jiang, Miao, Toshio, & Zhou, 2013). Wind tunnel tests found that wind erosion was greatly reduced when the plant cover was more than 30% (Scott Van Pelt et al., 2013). In our experiment, the cover under 40 and 80 mm treatments was 23% and 32% higher, respectively, than for the control. Such results imply that the role of ephemeral grasslands in the stabilization of sand dunes is strengthened by enhanced water input and would respond more highly in extreme precipitation years.

Second, in our research, water additions significantly increased the aboveground biomass of the plant community across the five study years (Tables 1 and 2). Increasing the biomass of individual species and the total net ecosystem primary productivity would supply fodder of higher nutritional quality for livestock production. In addition, aboveground net primary production (ANPP) is a key integrator of carbon uptake and energy flow in many terrestrial ecosystems, especially in desert ecosystems (Li, Zhang, et al., 2015). Dryland ecosystems account for approximately one‐third of the vegetation carbon in the world (Smith et al., 2000); vegetation carbon is sensitive to climate change and has been increased by the precipitation changes in Central Asia (Li, Zhang, et al., 2015; Smith et al., 2000). In addition, ephemerals can be divided into two types according to animal favor, unpalatable and palatable grasses (Anderson, Hoffman, & O'Farrell, 2010). Our results showed that both types were affected differently by water additions. The productivity of palatable grass per plot (e.g., *E. oxyrrhynchum*, *T. arcuata*, *A. linifolium*, and *H. pulchella*) increased, while the unpalatable grass (e.g., *C. physodes*) showed no change in shoot biomass per plot (Appendix 5). These results support the hypothesis that palatable grasses for animals are more competitive than unpalatable grasses (Moretto & Distel, 1997). The magnitude of the increase in shoot production with enhanced precipitation was more marked for *H. pulchella*, *T. arcuata* and *A. linifolium* than for other plants, suggesting higher water sensitivity for these three palatable grasses.

## 5 CONCLUSIONS

In the face of the global climate change, ephemeral populations and plant communities in desert ecosystems are likely to play critical roles in the stability of these fragile ecosystems. Our study showed that the growth of all plant species and the productivity of the communities increased markedly with enhanced water input. The response of plant community compositions to the increased precipitation was primarily reflected by changes in plant density, while increased precipitation did not affect the plant species richness and diversity index. Dominant species drove the response of plant density to increased precipitation in the five‐year study period. Moreover, increased precipitation had important effects on ecosystem functions. Increased precipitation increased the soil seed bank and altered its composition, as well as increased the productivity of palatable grasses for animals (e.g., *E. oxyrrhynchum*, *T. arcuata*, *A. linifolium*, and *H. pulchella*).

These findings have improved our understanding of how increased precipitation drives changes in plant community compositions in desert grasslands and will help us to better predict alterations in the community compositions of ephemerals under future global climate change scenarios. However, whether or how ecosystem functioning would be changed by changes in the precipitation regime in this desert ecosystem are still poorly understood, and more long‐term and field experiments need to be performed in the future to answer this question.

## CONFLICT OF INTEREST

The authors declare no conflicts of interests.

## AUTHOR CONTRIBUTIONS

Y.J. and G.F. conceived the ideas and designed methodology; Y.S., Z.S., T.Z., B.M., and C.T. collected the data; Y.J. and Y. S. analyzed the data; Y.J. and Y. S. wrote the article.

### Open Research Badges

This article has earned an Open Data Badge for making publicly available the digitally‐shareable data necessary to reproduce the reported results. The data is available at https://doi:10.5061/dryad.g1m5k70.

## Data Availability

Data available from the Dryad Digital Repository: https://doi:10.5061/dryad.g1m5k70.

## Appendix 1

Annual precipitation and soil physical and chemical properties of the experimental site. Values within a column followed by different letters are significantly different at *p* < .05.

**Table:**

Years	Annual precipitation (mm)	Ph (1:5 soil/water)	Organic matter (%)	Olsen‐ P (mg/kg)	Electrical conductivity (Ms/cm)
2005	141.6	7.87 ± 0.03b	0.18 ± 0.04ns	1.65 ± 0.03b	0.71 ± 0.08ns
2006	135.3	7.93 ± 0.02b	0.14 ± 0.01ns	2.25 ± 0.15a	0.89 ± 0.09ns
2007	231.2	8.26 ± 0.04a	0.14 ± 0.01ns	2.50 ± 0.11a	0.78 ± 0.10ns
2008	110.2	7.94 ± 0.03b	0.15 ± 0.02ns	2.31 ± 0.07a	0.79 ± 0.07ns
2009	166.2	8.01 ± 0.03b	0.14 ± 0.01ns	2.46 ± 0.08a	0.82 ± 0.08ns

## Appendix 2

The frequency of the plant species appeared in all 88 plots and the relative abundance of each species in community in 5 years (2005–2009). Letters in life type column represented annual plant (A) or perennial (P) plant; ephemeral (E) or nonephemeral plant; shrub (S) or herbaceous (H); *Dominant species and ^#^rare species in the experimental site.

**Table:**

Latin name of plant	Life type	Occurring frequency (%)	Relative abundance (%)
*Alyssum linifolium**	A,E,H	81	11
*Arnebia decumbens#*	A,E,H	23	1
*Astragalus* spp*.#*	A,E,H	27	0
*Carex physodes**	P,E,H	94	18
*Corispermum lehmannianum#*	A,E,H	48	2
*Eremopyrum triticeum #*	A,E,H	40	1
*Erodium oxyrrhynchum**	A,E,H	100	12
*Heliotropium ellipticum#*	A,E,H	2	0
*Hyalea pulchella**	A,E,H	85	5
*Hypecoum parviflorum#*	A,E,H	17	0
*Lappula* spp.	A,E,H	52	1
*Lithospermum arvense#*	A,E,H	2	0
*Nonea caspica#*	A,E,H	21	1
*Salsola praecox#*	A,E,H	29	1
*Senscio subdentatus#*	A,E,H	4	0
*Trigonella rcuata**	A,E,H	92	9
*Ceratocarpus arenarius**	A,E,H	96	15
*Schismus arabicus*	A,E,H	58	15
*Isatis* spp*.#*	A,E,H	2	0
*Eremopyrum orientale#*	A,E,H	2	0
*Nepeta micrantha*	A,E,H	50	4
*Nepeta pungens#*	A,E,H	25	1
*Echinops sphaerocephalus#*	A,E,H	4	0
*Amberboa turanica#*	A,E,H	6	0
*Horaninowia ulicina*	A,NE,H	67	3
*Soranthus meyeri#*	A,NE,H	6	0
*Allium* spp*.#*	A,NE,H	6	0
*Agriophyllum squarrosum#*	A,NE,H	27	0
*Euphorbia* spp*.#*	A,NE,H	23	0
*Ephedra distachya#*	P,NE,S	2	0
*Artemisia arenaria#*	P,NE,H	4	0
Compositae, unidentified to genus#	A,H	17	0
Compositae, unidentified to genus#	A,H	6	0
Chenopodiceae. Unidentified to genus#	A,H	8	0
Chenopodiceae. Unidentified to genus#	A,H	13	0
Unidentified 1#	A,H	6	0
Unidentified 2#	A,H	2	0

## Appendix 3

General view of the study site and experimental communities. (a) Plot diagram; (b) Field image. The experimental design includes two treatments: control and 40 mm water addition (only in 2006, one more treatment: 80 mm addition).

## Appendix 4

The monthly precipitation and air temperature of experimental site during the experimental period.

## Appendix 5

Response of aboveground biomass of dominant plant species to water input. Columns marked with different letters indicated significant differences (*p* < .05) between treatments. Error bars represented ± *SE*.
